# Global Proteomics-based Identification and Validation of Thymosin Beta-4 X-Linked as a Prognostic Marker for Head and Neck Squamous Cell Carcinoma

**DOI:** 10.1038/s41598-017-09539-w

**Published:** 2017-08-22

**Authors:** Li-Hsing Chi, Wei-Min Chang, Yu-Chan Chang, Yung-Chieh Chan, Chia-Chen Tai, Kam-Wing Leung, Chi-Long Chen, Alexander TH Wu, Tsung-Ching Lai, Yu-Chuan (Jack) Li, Michael Hsiao

**Affiliations:** 1 The Ph.D. Program for Translational Medicine, College of Medical Science and Technology, Taipei Medical University and Academia Sinica, Taipei, Taiwan; 2 Genomics Research Center, Academia Sinica, Taipei, Taiwan; 3 Division of Oral and Maxillofacial Surgery, Department of Dentistry, Taipei Medical University Hospital, Taipei, Taiwan; 4 Department of Density, Yuan’s General Hospital, Kaohsiung, Taiwan; 5 Departments of Density, Kaohsiung Veterans General Hospital, Kaohsiung, Taiwan; 6 Department of Pathology, Taipei Medical University Hospital, Taipei Medical University, Taipei, Taiwan; 7 Department of Pathology, College of Medicine, Taipei Medical University, Taipei, Taiwan; 8 Graduate Institute of Biomedical Informatics, College of Medicine Science and Technology, Taipei Medical University, Taipei, Taiwan; 9 Department of Biochemistry, College of Medicine, Kaohsiung Medical University, Kaohsiung, Taiwan

## Abstract

Head and neck squamous cell carcinoma (HNSCC) represents a major health concern worldwide. We applied the matrix-assisted laser desorption/ionization (MALDI) imaging mass spectrometry (IMS) to analyze paired normal (N) and tumor (T) samples from head and neck squamous cell carcinoma as well as liquid chromatography with tandem mass spectrometry (LC-MS/MS) analysis in HNSCC cell lines to identify tumor-associated biomarkers. Our results showed a number of proteins found to be over-expressed in HNSCC. We identified thymosin beta-4 X-linked (TMSB4X) is one of the most significant candidate biomarkers. Higher TMSB4X expression in the tumor was found by N/T-paired HNSCC samples at both RNA and protein level. Overexpression of TMSB4X was found significantly associated with poor prognosis of overall survival (OS, P = 0.006) and recurrence-free survival (RFS, P = 0.013) in HNSCC patients. Silencing of TMSB4X expression in HNSCC cell line reduced the proliferation and invasion ability *in vitro*, as well as inhibited the cervical lymph node metastasis *in vivo*. Altogether, our global proteomics analysis identified that TMSB4X is a newly discovered biomarker in HNSCC whose functions resulted in enhanced proliferation and metastasis *in vitro* and *in vivo*. TMSB4X may be a potential therapeutic target for treating HNSCC patients.

## Introduction

Head and neck squamous cell carcinoma (HNSCC), which is derived from the oral cavity, oropharynx and hypopharynx in more than 90%^1^, is the fifth leading cause of cancer death in Taiwan. The mortality rate of HNSCC patients in the male is 11.1-fold greater than that in women. The age-standardized incidence rate of HNSCC in males exceeds 30 in 100,000 populations in Taiwan^2^. Some factors, including virus infection, alcohol, carcinogens in betel quid and tobacco, physical irritations, or host susceptibility, may contribute to HNSCC^3, 4^. The modern treatment of HNSCC should be done by surgery alone, radiation therapy alone, or a combination of them with adjuvant chemotherapy according to National Comprehensive Cancer Network (NCCN) guidelines^5^. Despite those interventions, the 5-year survival rate for this disease has improved only marginally over the past decade, and recurrent disease is observed in 50% of all patients^1, 6^. By comparison with new systemic treatments seen in other solid tumors, the prognostic result of targeted therapy, ex. EGFR inhibitors, in HNSCC patients has remained relatively stationary in recent years^7^. Patients with early-stage cancer often manifest minimal physical findings, which usually ignored by themselves, resulting in delayed diagnosis regarding poor tumor control and patient survival. Therefore, quitting bad habits, routine screening and early intervention will be helpful to improve their survival while maintaining the quality of life. The key advancement of those interventions should be (i) the discovery of high impact prognostic biomarkers, (ii) and planning of the proper treatment modalities for accuracy sub-grouped HNSCC patients.

Recently, proteomics analysis is a promising approach to the identification of proteins with various abundance related to the prognostic categorisation of cancer^8^. MALDI IMS generates profiles and two-dimensional ion density maps of molecules, which are primarily peptides or proteins, directly from the surface of thin tissue sections of HNSCC^9^. These data give the relative abundance of the molecules with their spatial distribution in tissue samples. Comparative proteomics analysis by using LC-MS/MS analysis to identify the differentially expressed proteins in HNSCC was also reported in previous articles^10, 11^.

Here we used both MALDI IMS and LC-MS/MS for the biomarker mining. In consensus candidates from these two modalities, TMSB4X has lowest expression level in normal oral mucosa, whereas it is highly abundant in late-stage HNSCC samples. Then, we used immunohistochemistry (IHC) staining to confirm the expression level of endogenous TMSB4X with clinicopathological survival analysis. Furthermore, we knocked down TMSB4X in HNSCC cell line and found that tumor growth and metastasis is attenuated. Taking all together, through multiple proteomics approaches we elucidated that TMSB4X might be a new candidate for HNSCC therapy.

## Results

**Figure 1:**
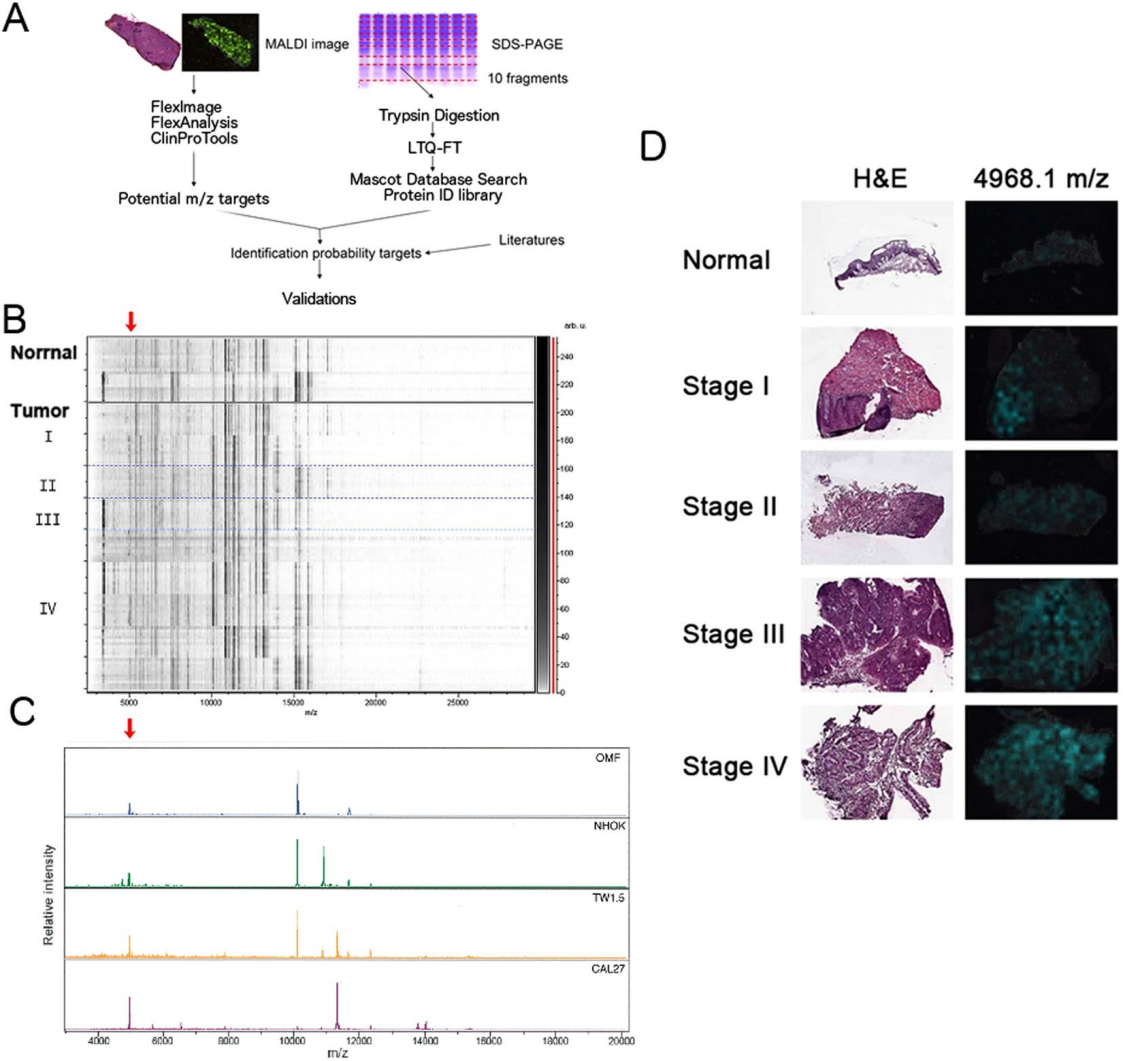
Identify the biomarker through MALDI imaging mass spectrometry (MALDI IMS). (**A**) Overview of the strategy used for identification of the biomarkers of HNSCC. (**B**) Profiling of stage I, II, III and IV of HNSCC proteomes from MALDI IMS by the ClinProTools analysis. (**C**) Peaks found in Normal (OMF and NHOK) and cancer cells (TW1.5 and CAL27). (**D**) The left panel represented the histomorphology of clinical HNSCC samples subject to MALDI IMS analysis after H&E stain. The right panel showed the intensity distribution of 4968.1 m/z, which was stronger in advanced stages. Red arrow: the intensity of T > the intensity of N.

We utilized a bottom-up strategy to determine whether it is possible to find new prognostic marker(s) in HNSCC patients (Fig. 1A) in a step-wise approach. Frozen serial sections of freshly preserved HNSCC specimen were subjected to histopathological examinations and MALDI-IMS analysis. The protein identification database from protein lysates of several HNSCC cell lines and normal oral cell lines was then established by using one-dimension-gel LC-MS/MS proteomics analysis. After the comparison of the datasets from MALDI-IMS and LC-MS/MS, primary candidates were identified and selected after literature reviews. The picked candidate proteins were stained in HNSCC patient cohort to determine the prognostic values. *In vitro* functional analysis of cellular proliferation, and invasion/migration in HNSCC cell lines as well as *in vivo* metastasis assays were then performed to determine the selected gene functional effects in HNSCC cells.

### The MALDI imaging mass spectrometry (MALDI IMS) and statistics analysis obtained from HNSCC and normal specimens

**Figure 2:**
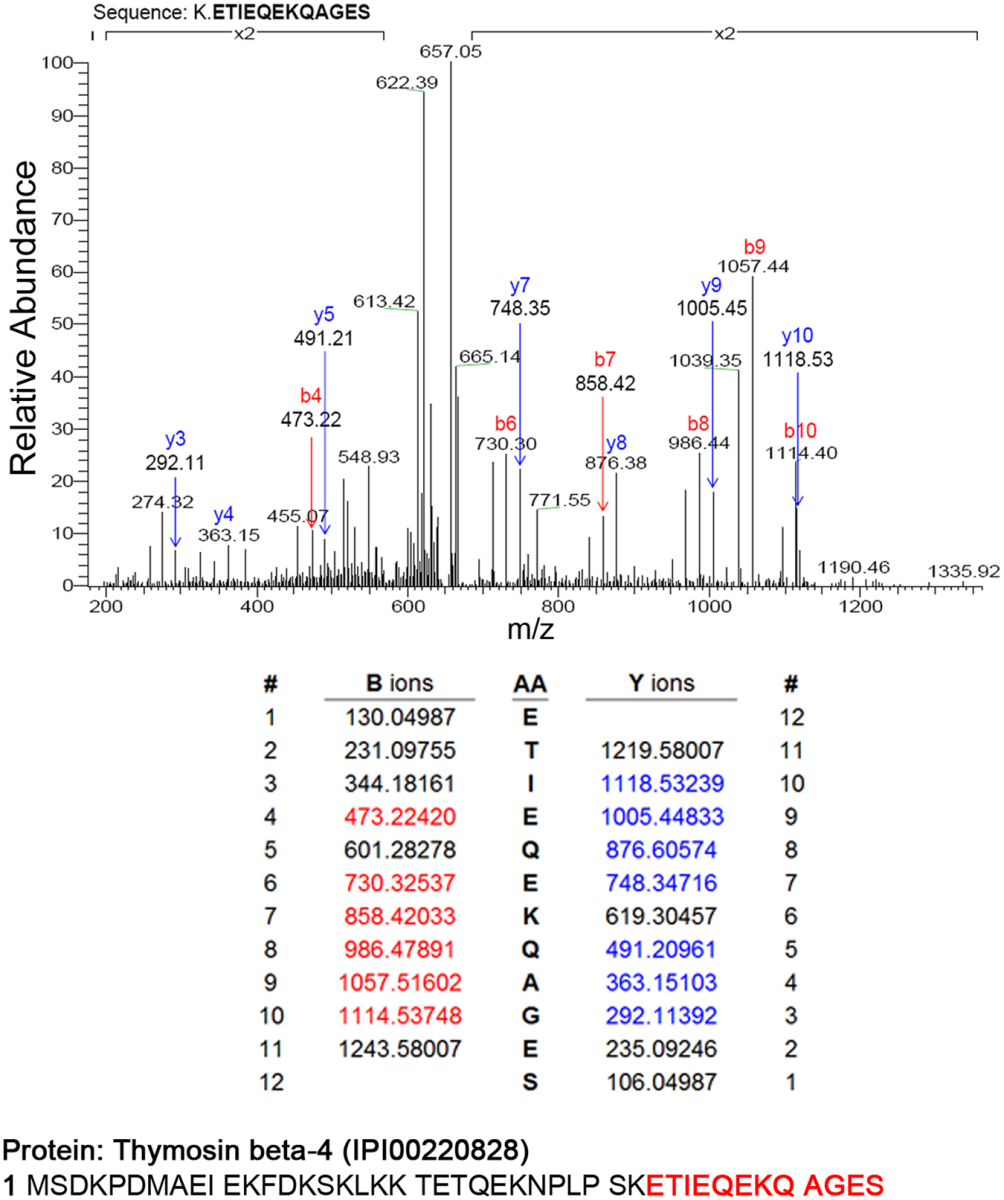
LC-MS/MS analysis of TMSB4X. The data showed sequencing of the trypsinized peptide K.ETIEQKQAGES, which was identified as TMSB4X in CAL27 cell line. Peptide sequencing was indicated by matching B-ions (red) and Y-ions (blue) fragments. B-ions: the charge is retained by the amino-terminal part of the peptide. Y-ions: the charge is retained by the carboxyl-terminal part of the peptide.

Eleven fresh-frozen samples were examined using the MALDI IMS, including two normal oral mucosal specimens and nine HNSCC specimens from two cases of stage I, one case of stage II, one case of stage III and five cases of stage IV HNSCC, as our discovery cohort. Cancer and non-cancerous part were defined by a pathologist under H&E staining of the first section. Ten mass spectra per spot in tissue slides were obtained for protein profiling and processed by the ClinProTools software (Bruker Daltonics, Billerica, MA). The analysis was performed according to the cutoff by the P value of the t-test/ANOVA (PTTA) less than 0.05. The small-sized molecules were easily to be vaporized and captured by MALDI-TOF/TOF system than by LC-MS/MS system^12–14^. Thus, we found that the peak spectra presented from 3,000 to 20,000 m/z (Fig. 1B). There were about 200 significant signals of the mass spectra were picked up corresponding to abundant presented in tissue sections. We chose the 48 considerably differential expressed candidates between tumor and normal tissue by peak statistics (Table 1), which the T/N ratio of intensities is larger than 1. Then we zoomed in the top 10 candidates. According to the mass of candidates, we tried to match their protein identifications by comparing the MALDI-MS of normal cell lines (Oral Mucosa Fibroblast, OMF, and Normal Human Oral Keratinocyte, NHOK) and cancerous cell lines, TW1.5 and CAL27 (please refer to Fig. 1C). There was a peak at 4966.6 m/z, which had the highest intensity in CAL27 cells than in normal cells. The 2-dimensional MALDI images of an intensity distribution of peaks were presented and compared with its counter partner under H&E stain (data processed by using flexImaging software). With the references to the MALDI images, we also noticed that a peak 4968.1 m/z, from the top 10 candidates, had much higher intensity in stage III or IV than in stage I, II or normal tissue (Fig. 1D). Thus, we performed the LC-MS/MS amino acid sequencing of the 1D-gel of CAL27 cell lysates with the similar molecular weight, an abundant peptide sequence, K.ETIEQKQAGES, was identified as IPI00220828, International Protein Index of TMSB4X (Fig. 2). By comparison of the MALDI-IMS protein candidates listed in Table 1, the peak at 4968.1 m/z could be identified as TMSB4X. We proposed, this candidate, TMSB4X, presented the positive correlation between its signal intensity and the cancer stage in the MALDI images. It encouraged us to further investigate the TMSB4X as a potential biomarker.

**Table 1:** Peak statistics (candidates listed with T/N > 1).

Mass	PTTA	Ave_N	Ave_T	Std_N	Std_T	T/N
10099.8	7.98E-16	341.9	1509.6	188.2	1009.9	4.415
10900.9	3.64E-17	113.6	425.3	40.6	253.0	3.744
4048.6	7.34E-13	18.4	47.7	3.5	30.5	2.597
11659.2	1.17E-07	300.6	733.7	222.4	321.3	2.441
10306.9	1.86E-15	104.2	247.2	34.7	111.2	2.373
9756.5	2.75E-13	31.4	71.6	8.1	38.8	2.277
4968.1	0.0147	28.0	62.8	14.0	109.8	2.241
11731.0	0.000000000183	69.9	150.2	27.1	78.6	2.148
11370.2	0.00000394	576.2	1237.7	362.1	828.0	2.148
10532.3	0.000000000000000772	48.4	96.7	11.6	35.8	2.000
22696.7	0.000282	10.5	20.7	4.5	19.2	1.910
22777.9	0.00000208	10.5	19.7	5.3	9.6	1.88
8459.8	0.000000106	39.7	74.6	12.8	45.5	1.877
5048.9	0.00000000118	94.1	175.6	19.2	99.2	1.867
4937.5	0.000778	14.4	26.6	5.2	27.2	1.845
17892.5	0.00000593	12.7	23.2	6.1	12.7	1.828
27819.3	0.00126	8.8	15.7	4.3	14.6	1.779
5449.7	0.0000000539	37.3	65.9	13.8	25	1.766
9850.0	0.00000000498	15	24.9	4.1	9.8	1.666
5828.8	0.0000709	86.1	134.7	35.6	52.6	1.564
4328.4	0.000247	20.4	31.8	6.8	20.7	1.561
10750.8	0.0000051	37.7	58.5	12.7	21.2	1.552
5685.1	0.000778	107.8	165.1	40.9	105	1.531
16799.2	0.00103	36.3	54.7	15.4	29.3	1.507
12698.5	0.00438	582.2	874.3	297.6	471.2	1.502
6123.7	0.00000000118	58.5	87.8	10.9	29.1	1.502
13238.6	0.0082	220.6	323.9	110.3	192.5	1.468
9377.6	0.0000276	37.6	54.9	9.9	25.4	1.459
5151.1	0.00000622	43.2	62.8	10.7	26.4	1.455
8572.3	0.0187	136.6	196.1	70.4	132.5	1.436
9627.0	0.000000477	40.7	58	8	21.6	1.425
13162.6	0.0316	1249.8	1778.5	778.9	947.3	1.423
13372.6	0.041	207	284.5	117.7	158.2	1.375
8408.0	0.0025	59.9	82.1	21.9	31.9	1.372
11834.7	0.0147	75.9	102.9	35.4	36.1	1.356
3584.5	0.018	3.8	5.1	1.5	3.1	1.356
8649.1	0.0191	68.3	90.8	29.4	41.1	1.329
6349.4	0.0105	183	242	71.9	84.8	1.323
5361.5	0.00732	92	120.3	27.8	57.5	1.307
8966.4	0.0237	65.4	85.4	29.4	24.1	1.305
10453.9	0.00151	43.4	56.6	8.4	28.2	1.302
11988.4	0.0411	48.8	63.5	23.5	24.1	1.301
12908.6	0.0105	80.8	104.7	23.7	54.1	1.296
9161.8	0.0328	24.6	31.4	10.7	9	1.277
8190.8	0.0187	44.6	56.6	16.4	16.5	1.268
4133.2	0.0246	8.8	11	2.8	4.9	1.251
5269.0	0.018	34.7	41.4	8.5	12.6	1.195
5935.0	0.0274	23.6	28	6.1	8.7	1.186

T_Std: the mass standard deviation of tumor specimen, T_Ave: the averaged intensity of tumor specimen; N_Std: the mass standard deviation of normal tissue, N_Ave: the averaged intensity of normal tissue; PTTA: the P value of the t test/ANOVA of the individual peak; T/N: the ratio of Ave_T/Ave_N.

### Establishment of the protein databases of normal and HNSCC cell lines by 1D gel LC-MS/MS

To facilitate the candidate selection from MALDI IMS dataset, we used one-dimension-gel LC-MS/MS method to prepare a protein library. Total protein was extracted from several cell lines derived from HNSCC, which include Ca9-22, CAL27, HSC-3, SAS, SCC-4, TW1.5, TW2.6, as well as normal oral keratinocytes (NHOK) and oral mucosa fibroblasts (OMF). There were 39,941 to 44,445 peptides were found in each cell lines. Eventually, 1,210 proteins were found in NHOK while 1,286 proteins were revealed from OMF. There were 1,198–1,603 proteins identified in these 7 HNSCC cell lines. In the Venn diagram among these three protein datasets (Supplementary Figure S1), there were 30 proteins were exclusively identified among all HNSCC cell lines rather than within NHOK and OMF (Supplementary Table S1). Thus, we provided a list of HNSCC cancer-specific proteins from multiple cancer cell lines.

### TMSB4X was highly expressed in tumor samples than in paired normal tissues

**Figure 3:**
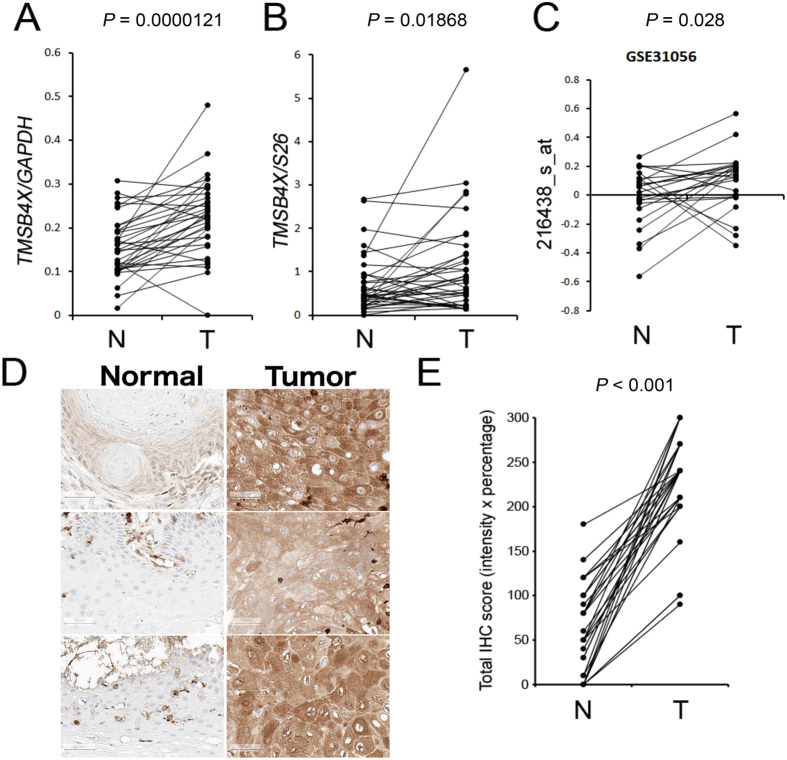
TMSB4X has higher RNA or protein expression level in tumor samples than in normal adjacent tissues, which was demonstrated in 3 independent HNSCC cohorts. (**A**,**B**) Showed the expression changes of TMSB4X in 35 NT-paired samples of KVGH cohort by RT-PCR and real-time PCR, respectively. (**C**) TMSB4X (216438_s_at) expression in 23 NT-paired HNSCC cohort in microarray dataset, GSE31056. (**D**) IHC staining in tissue microarray from 31 NT-paired HNSCC samples of TMUH cohort, representative 3 NT-paired images. (**E**) TMSB4X IHC expression was scored by multiplying intensity and percentage. The P value was calculated by paired t-test.

The expression level of TMSB4X in HNSCC was objected to being validated by an RT-PCR and real-time PCR in 35 pairs of the fresh-frozen HNSCC samples (T) and adjacent non-tumor tissues (N), and from 23 NT-paired dataset from the public HNSCC microarray cohort (GSE31056). In our MALDI IMS analysis, the signal at m/z 4968.1 (TMSB4X) showed 2.2-fold change (PTTA = 0.0147) in the HNSCC tissues than in the normal tissues (Table 1). Furthermore, the RT-PCR (Fig. 3A) and real-time PCR results (Fig. 3B) of NT-paired samples, TMSB4X also presented about 1.48 (P < 0.00001) and 1.59 (P = 0.01868) fold change, respectively. In the GSE31056 dataset, TMSB4X (probe 216438_s_at) expression was higher in T than in N (P = 0.028) (Fig. 3C). Subsequently, in immunohistochemistry staining of 31 NT-paired HNSCC tissue microarray, TMSB4X was significantly elevated (P < 0.001) in T than in N (Fig. 3D and E).

### High expression of TMSB4X is significantly associated with poor overall and recurrence-free survivals in HNSCC patients

The results mentioned above that the expression of TMSB4X is high in HNSCC at both RNA and protein levels, it encouraged us to elucidate TMSB4X as a prognostic biomarker. TMSB4X was overexpressed and behaved as a biomarker in colorectal cancer (CRC)^15–18^. On the slides of tissue microarray of our HNSCC patient cohort, the dots were organized with the human specimens as well as the mice xenografts derived from several kinds of human cell lines in order to ensure the quality control of TMSB4X IHC staining. The images of CRC xenografts on our slides were shown in Supplementary Figure S2(A,C,D). We also used isotype antibody control IgG to perform the IHC staining on the slides to acquiring the negative control (Supplementary Figure S2B) The resulting images could make a guarantee of the quality in our IHC staining.

**Figure 4:**
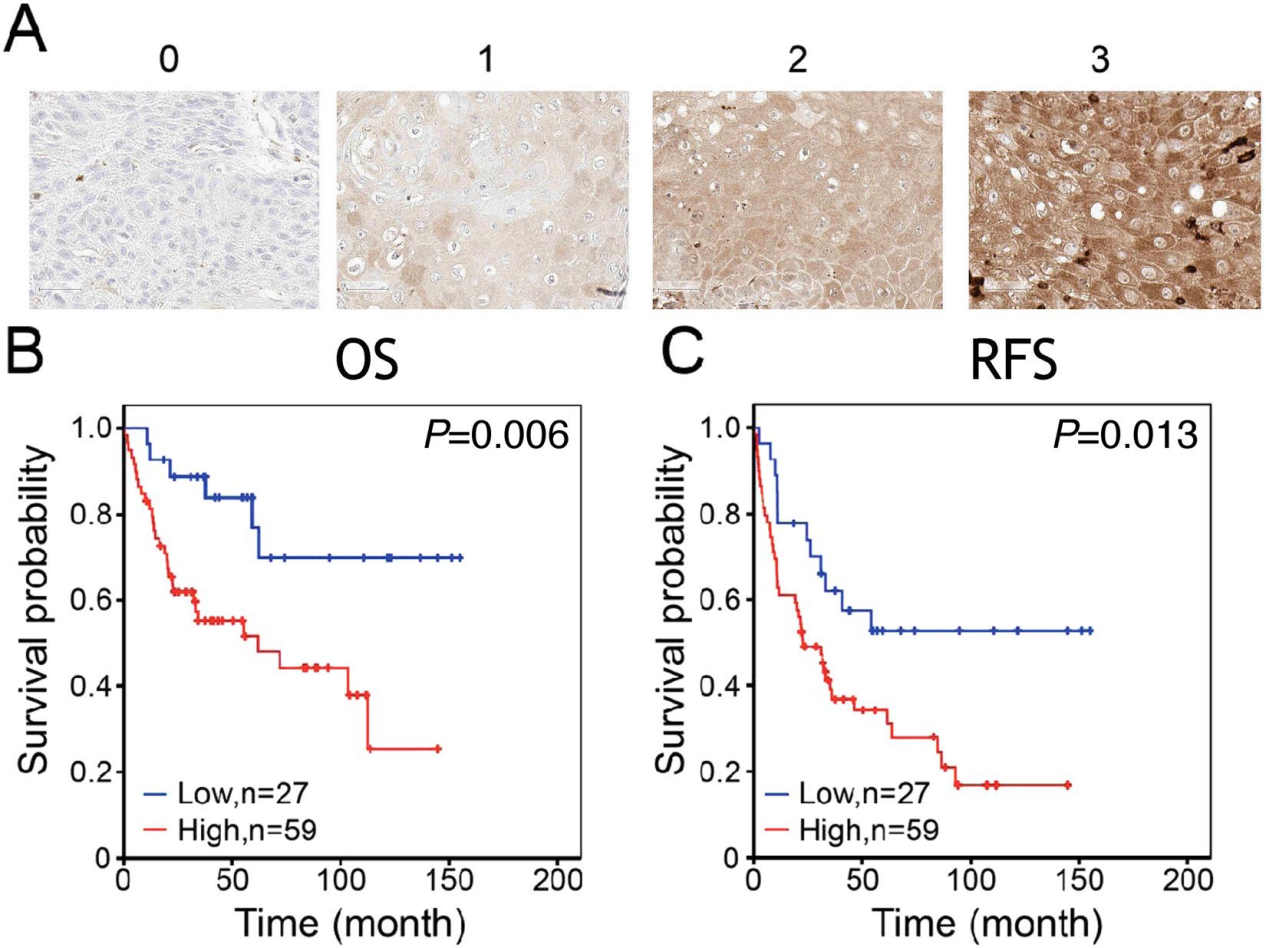
Higher TMSB4X IHC expression is correlated with poor prognosis in HNSCC cohort. (**A**) The representative images with different IHC intensity of TMSB4X. Scale bar represents 50 µm. (**B**,**C**) Kaplan-Meier estimates of OS (*P* = 0.006) and RFS (*P* = 0.013) were categorized by TMSB4X level. Patients with high TMSB4X had significantly poor prognosis. Statistics was calculated by log-rank test.

The HNSCC patients were categorized by the IHC scores at the cutoff value (median) of 240. In the correlation analysis of clinicopathological features, the patients with high IHC expression of TMSB4X were found significantly associated with tumor recurrence (P = 0.017) (see Table 2), and also presented poor overall survival rate (OS, P = 0.006, Fig. 4B) as well as recurrence-free survival rate (RFS, P = 0.013, Fig. 4C). We performed Cox proportional hazards regression analysis to calculate the prognostic impact of TMSB4X overexpression. The univariate survival analysis showed higher T status, N status or TMSB4X expression were significantly associated with adverse outcome in both OS and RFS (see Table 3, the hazard ratios (HR) are greater than 1.89 or above). In the multivariate survival analysis adjusted with T status and N status, it revealed that TMSB4X expression is an independent prognostic factor for OS and RFS in HNSCC patients. Therefore, we may infer within 95% confidence interval (CI) that the OS from high TMSB4X expression is approximately 3.12 times, and at least 1.27 times, the risk from low expression one. (HR = 3.12, 95% CI = 1.27–7.63, P = 0.013), as well as the RFS from high TMSB4X expression is around 2.07 times, and at least 1.08 times, the danger from low expression one. (HR = 2.07, 95% CI = 1.08–3.98, P = 0.028)

**Table 2:** The correlation analysis of TMSB4X expression and clinicopathological features in HNSCC cohort.

Features	Case no	TMSB4X expression				*P* value
		Low(n = 27) (%)		High(n = 59) (%)		
Age at diagnosis						
<65 y	70	22	31.4%	48	68.6%	0.989
>=65 y	16	5	31.3%	11	68.8%	
Gender						
Male	79	24	30.4%	55	69.6%	0.495
Female	7	3	42.9%	4	57.1%	
T Status						
T1 + T2	60	22	36.7%	38	63.3%	0.110
T3 + T4	26	5	19.2%	21	80.8%	
N Status						
N0	59	18	30.5%	41	69.5%	0.793
N1-N3	27	9	33.3%	18	66.7%	
M Status						
M0	84	27	32.1%	57	67.9%	0.333
M1	2	0	0.0%	2	100.0%	
**Clinical Stage**						
Stage I + II	45	15	33.3%	30	66.7%	0.685
Stage III + IV	41	12	29.3%	29	70.7%	
Recurrence						
No	32	15	46.9%	17	53.1%	0.017*
Yes	54	12	22.2%	42	77.8%

**Table 3:** Univariate/Multivariate Cox proportional hazards regression analyses on OS and RFS time of TMSB4X gene expression in HNSCC.

Features		Univariate analysis of OS			*P* value	Multivariate analysis of OS			*P* value
		HR	95% CI (L)	95% CI (H)		HR	95% CI (L)	95% CI (H)	
**T Status**									
	T1 + T2	1				1			
	T3 + T4	3.22	1.67	6.23	0.001*	2.62	1.31	5.23	0.006*
**N Status**									
	N0	1				1			
	N1-N3	2.06	1.06	4.01	0.033*	1.73	0.87	3.45	0.121
**TMSB4X**									
	Low	1				1			
	High	3.26	1.35	7.89	0.009*	3.12	1.27	7.63	0.013*
**Features**	**Univariate analysis of RFS**				***P*** **value**	**Multivariate analysis of RFS**			***P*** **value**
		**HR**	**95%CI**	**95%CI**		**HR**	**95%CI**	**95%CI**	
**T Status**									
	T1 + T2	1				1			
	T3 + T4	1.89	1.08	3.31	0.027*	1.56	0.87	2.79	0.132
**N Status**									
	N0	1				1			
	N1-N3	1.74	0.99	3.04	0.050	1.61	0.91	2.83	0.101
**TMSB4X**									
	Low	1				1			
	High	2.21	1.16	4.21	0.016*	2.07	1.08	3.98	0.028*

*P < 0.05 (Cox proportional hazards regression analyses). The 95% confidence interval (CI) for the hazard ratio (HR) in overall survival (OS) or recurrence-free survival (RFS).

### Knockdown of TMSB4X inhibits cell proliferation and invasion *in vitro*, as well as neck LN metastasis *in vivo*

**Figure 5:**
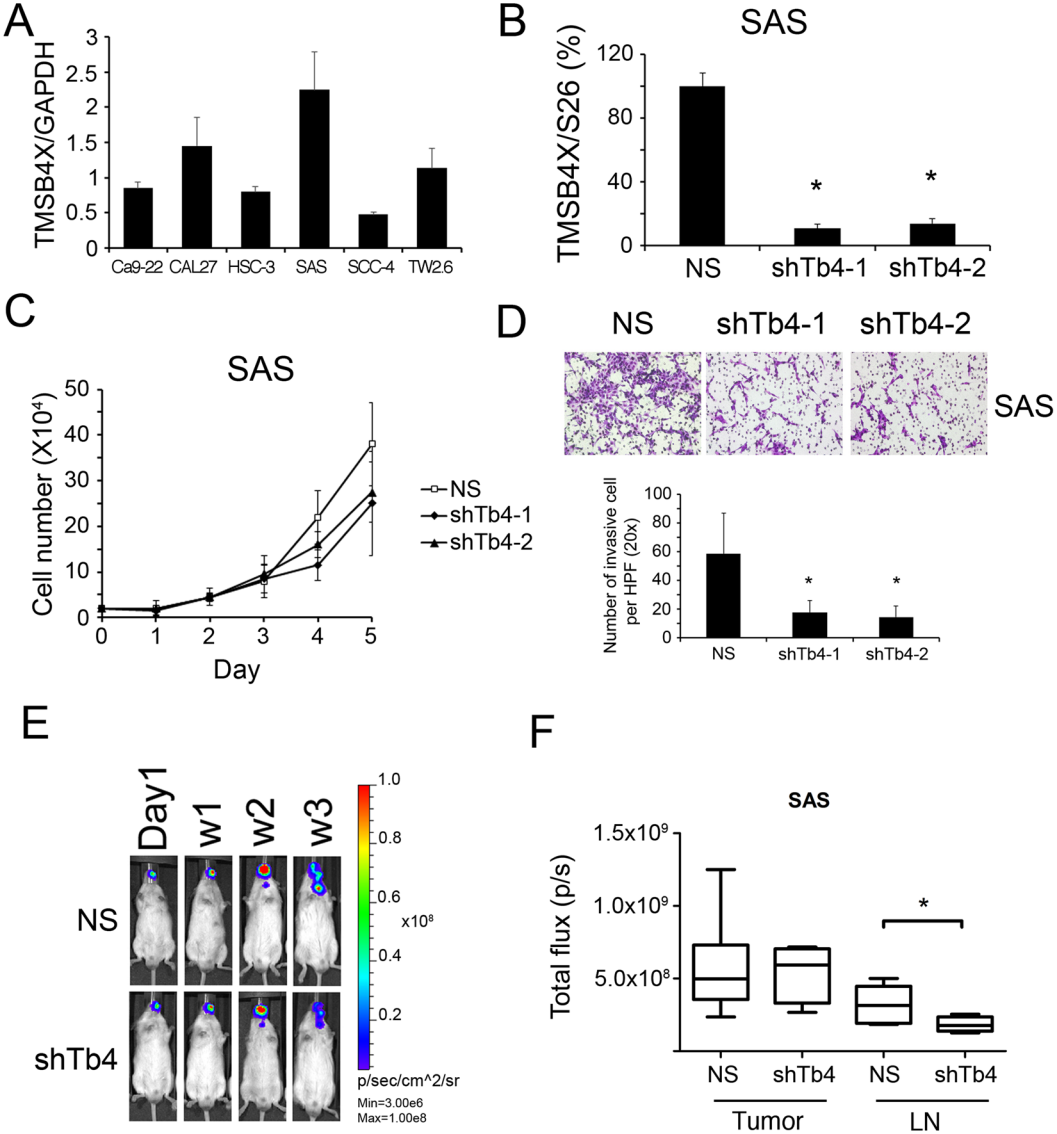
Cell function assays. Knockdown TMSB4X in SAS cells may inhibit their cell proliferation and invasion ability *in vitro*, as well as suppress cervical LN metastasis *in vivo*. (**A**) The panel of TMSB4X expression level in 6 HNSCC cell lines by RT-PCR. The relative aggressive SAS cells have abundant TMSB4X. (**B**) In TMSB4X knockdown clones of SAS, the expression was revealed by RT-PCR. (**C**) The growth curves of TMSB4X knockdown clones of SAS. (**D**) The invasion ability of TMSB4X knockdown clones of SAS was performed by Boyden chamber assay. Cell photography and number were taken and counted at 200x magnification (high power field, HPF). (**E**) Bioluminescence images were taken from the buccal orthotopic xenograft mouse model. The total flux of bioluminescence was measured separately at mouse buccal (Tumor) and neck lymph node (LN metastasis). (**F**) The box-plot of LN metastasis was drawn according to the total flux value in region of interest (ROI). Asteroid *denotes *P* < 0.05. Statistics was calculated by student t-test. (shTb4-1: clone1 with shRNA-TMSB4X).

To test the functions of TMSB4X in HNSCC, we surveyed the mRNA expression of TMSB4X among HNSCC cell lines (Fig. 5A). We applied lentiviral shRNA successfully to suppress TMSB4X in SAS, which has most abundant endogenous TMSB4X (Fig. 5B). In cell functions assays, there is 32–40% reduction of cell proliferation (Fig. 5C). It has been reported that TMSB4X may enhance cancer metastasis^19^. We further determined the migration and invasion ability of each clone. Knockdown TMSB4X in SAS cells could reduce their invasion ability (Fig. 5D). The proliferation of SAS cells as well as cervical lymph node metastasis in orthotopic mice model (left side buccal inoculation) were determined by the bioluminescence from IVIS image. There is no significant difference of *in vivo* growth curve between those cells on the primary sites of buccal mucosa. There is signal attenuation in week three due to the wound scab which blocked the transmission of light. Furthermore, the knockdown clone of TMSB4X exhibited lesser signal at the neck, which implied the inhibition of ipsilateral cervical metastasis of SAS cells (Fig. 5E).

## Discussion

Direct analyzing fresh frozen tissues with the label-free MALDI IMS technology could identify potential disease biomarkers. It has been used in many research fields such as metabolomics, drug discovery, cancer biomarkers, molecular pathological diagnosis^20–23^. There is an issue about sample contamination by saliva or tear which contains secretory TMSB4X^24^, we should take care of MS samples handling in all proteomics analysis.

Previous researchers who used the MALDI IMS alone could find many different peaks between normal and tumor tissue in HNSCC but rarely identify protein targets^25^. In our study, there is an abundant peak found in tumor tissue around 4968.1 m/z, and it is much more in the stage III or IV than in stage I or II. We established a protein library from several HNSCC cells for recognizing those candidates by corresponding mass from spectra. However, there are relatively restricted spectra (3,000 to 20,000 m/z) to be found in high-resolution MALDI IMS thus limited proteins could be identified. According to our results, TMSB4X expression is apparently up-regulated in HNSCC especially in the advanced stages of samples. We validated and confirmed the expression of TMSB4X in clinical HNSCC samples by IHC staining (Fig. 4) as well as in HNSCC cell lines (Fig. 5A) by RT-PCR. Furthermore, knockdown of TMSB4X in HNSCC cell line, SAS, could reduce the cell proliferation (Fig. 5C), invasion ability *in vitro* (Fig. 5D) as well as lymph node metastasis *in vivo* (Fig. 5E).

Thymosin beta-4 X-Linked (encoded by the TMSB4X gene), a 5053 Dalton protein containing 44 amino acids, is a kind of actin-sequestering peptides. It binds with monomeric globular actin and prevents its polymerization^26–28^. TMSB4X is tied up the actin monomers until cell signals trigger filament formation^27, 28^. It has been observed to be upregulated in various cancers, including colorectal, gastric and pancreatic cancers^18, 29, 30^. Gene expression analysis from Microarray database in cell lines from human primary and metastatic tongue squamous cell carcinoma showed that TMSB4X gene is expressed at about 4-fold higher in metastatic cells than in primary cells^31^. The previous study suggested that TMSB4X may regulate cell motility and metastasis in other human cancers as well as in mouse fibrosarcoma^32^. TMSB4X was reported to stimulate human tumor growth and metastasis^33^ by induction of cell invasion^34^ and vascular endothelial growth factor (VEGF)-mediated angiogenesis^33, 35–37^. These reports are compatible with our findings in this study.

The mechanism of TMSB4X to facilitate cancer cell motility was suggested through epithelial-mesenchymal-transition (EMT) in hepatoma as well as in HNSCC^34, 38^. Previously, Huang *et al*. found that TMSB4X overexpression in colorectal cancer may promote tumor progression by inducing an EMT via activation of integrin-linked kinase (ILK) and consequentially its signal transduction through phosphorylation of Akt, glycogen synthase kinase 3 (GSK3) and beta-Catenin^19, 39^. The nuclear entry of TMSB4X was also reported^40–43^ and associated with cancer cell migration^40^ via some upregulated genes and their nuclear co-localization. However, there was no obviously prognostic impact by the nuclear localization of TMSB4X in our HNSCC patient cohort (please see the supplementary Figure S3 and Table S2).

Controversially, the peptide segment ^17^LKKTETQ^23^, which is the active site within the TMSB4X protein, is the main ingredient in drug TB-500. TB-500 is claimed to promote actin binding, skin keratinocyte migration, collagen deposition and decrease inflammation regarding accelerating of wound healing. After that, this injectable drug is used for doping in human or equine sports^44^. In conclusion, TMSB4X is presented in fresh-frozen HNSCC tissues and can be detected by bottom-up approach via the MALDI IMS system combined with a protein library from HNSCC cell lines collected by LC-MS/MS system. We confirmed the association of TMSB4X on proliferation and metastasis of HNSCC *in vitro* as well as *in vivo*. TMSB4X could be served as a therapeutic candidate in the future anticancer research, ex. 1) TB-500 in conditioned culture medium to investigate the phenotype changes among HNSCC *in vitro*, or 2) to develop an antibody against ^17^LKKTETQ^23^ peptide to inhibit HNSCC progression. Recent advancement of the MALDI IMS technology has facilitated the analysis of formalin-fixed-paraffin-embedded (FFPE) tissue samples^45^. We will be encouraged to perform gene network analysis of HNSCC tissue microarray by this kind of approach.

## Materials and Methods

### Cell lines and cell culture condition

Seven HNSCC cell lines were employed in this study including Ca9-22, CAL27, HSC-3, SAS, SCC-4, TW1.5, and TW2.6. These cell lines were cultured in DMEM or DMEM/F12. All mediums supplemented with 10% (v/v) FBS (Gibco), 2 mM glutamine (Gibco), 100 units/mL penicillin (Gibco), and 100 mg/mL streptomycin (Gibco). All cell lines were incubated in a 37 °C humidified atmosphere (95% air and 5% CO_2_). The normal human oral keratinocyte (NHOK) was cultured in Keratinocyte-SFM (Gibco) and oral mucosa fibroblast (OMF) was cultured in DMEM. These two types of normal primary cultured cells were used as normal controls for all of our experiments.

### Patients

Eighty-six samples derived from HNSCC patients were obtained at Taipei Medical University Hospital (TMUH) in Taiwan from 1991 to 2010. Patients with preoperative chemotherapy or radiotherapy were excluded. All cases were classified according to the staging manual of American Joint Committee on Cancer (AJCC). The survival data were available in all cases, and the maximal observation period is 190 months. The overall survival (OS) and recurrence-free survival (RFS) were defined as the intervals between surgery to death and to recurrence to be diagnosed, respectively. The samples were collected with patient personal information delinked and under the approval of the Institutional Review Boards of Taipei Medical University (TMU-IRB 99049). Thirty-five normal and tumor (NT) paired HNSCC RNA samples were collected at Kaohsiung Veterans General Hospital during 1999 to 2001 with patient personal information delinked and carried out in accordance with the regulations and the institutional guidelines.

### Protein lysates preparation, SDS-PAGE and in-gel digestion

Cells were harvested and washed by 1x PBS on 10-cm culture dish then were lysed in 80 µL protein lysis buffer (20 mM Imidazole-HCl at pH 6.8, 2 mM MgCl_2_, 1 mM NaF, 1 mM sodium vanadate, 1 mM sodium molybetadate, 100 mM KCl, 20 mM EDTA at pH7.0, 30 mM sucrose, 50 µg/mL leupeptin, 30 µg/mL aprotinin, 1 mM phenylmethylsulfony fluoride) shaked on ice for 30 min and then centrifuged at 13,000 rpm for 30 min. All protein lysates were aliquot and stored at −80 °C.

Acrylamide gel was prepared under manufacture’s recommendation (Bio-Rad). A 50 µg total protein of each sample was heated in 4X sample buffer at 95 °C for 10 min and then loaded into each well of gel. Proteins were separated under 15% SDS-polyacrylamide gel electrophoresis (SDS-PAGE) for 30 min at 80 V and 120 min at 120 V. Then gel was stained with Coomassie Blue (J.T.Baker) for 10 min, followed by cut into small strip and washed once with ddH_2_O. The gel pieces were destained with 50% acetonitrile (ACN)/25 mM ammonium bicarbonate overnight at 4 °C. Each part of gel was stored in ddH_2_O at 4 °C.

The liquid containing gel was discarded and replaced with reduction buffer (10 mM DTT/25 mM ABC) for 1 hr at 56 °C. Discarded the reduction buffer and added alkylation buffer (55 mM IAA/25 mM ABC) for 1hr at room temperature where in the prevention of light. The solution was discarded and washed twice with 40% ACN for 10 min and then dehydrated by treatment with 100% ACN. The gel pieces were dried in vacuum and rewet with 0.12 µg of modified trypsin (Promega) in 25 mM ABC and digested overnight at 37 °C. Peptides in solution were transferred to new eppendorf tubes and extracted by using two 100 µL portions of 60% ACN/0.1% trifluoroacetic acid (TFA). The solution was dried in vacuum, and peptides were redissolved in 0.1% TFA for LC-MS/MS analysis.

### LC-MS/MS analysis

LC-MS/MS analyses were performed on a linear ion trap (LTQ) tandem mass spectrometer (LTQ-FT LC/MS/MS, Thermo Electron). The Mascot search engine (Matrix Science) was used for protein identification according to a curated protein database (IPI human, International Protein Index, compiled by the European Bioinformatics Institute). Peptide mass tolerance and fragment mass tolerance were set at 100 ppm and 0.25 Da, respectively. Proteins were scored using a probability-based MOWSE (for MOlecular Weight SEarch) algorithm, and the Mascot scores were reported in the form of −10 × log(P), where P is the probability that the observed match is a random event. The acquired data was searched against the International Protein Index (IPI) human protein sequence database by using the automated database-searching program, Mascot (Matrix Science). Spectra were searched with a mass tolerance of 15 ppm for MS data and 0.8 Da for MS/MS data. Up to two missed trypsin cleavages was allowed. Carbamidomethyl cysteine was set as a fixed modification, and oxidized methionine and deamidation was set as variable modifications. All identified proteins were summarized and exported as spread sheet in .xlsx file format.

### Tissue sample preparation for the MALDI IMS analysis

The fresh frozen tissue samples were maintained with the desired orientation on the cutting block using OCT (Optimum Cutting Temperature) polymer. A thin frozen section was cut at −15 °C by using a cryostat (Thermo Electron) and deposited on cold ITO-coated conductive glass slides (Bruker Daltonics). The slide and tissue section were then quickly warmed, which let the tissue thaw-mounted onto the slide. Tissue sections were fixed by immersion in 70% then 95% ethanol bath each for 30 sec and allowed to dry for 40 min in a desiccator. Matrix deposition was performed manually with a pipette. Two drops of 0.5–1 µL (the optimum volume depended on slide size) of freshly prepared 40 mg/mL SA dissolved in 50% ACN, 0.1% TFA was deposited onto each tissue slide.

### The MALDI imaging mass spectrometry (MALDI IMS) Imaging and Data Analysis

Imaging spectra were acquired on an UltraFlex MALDI-TOF/TOF instrument (Bruker Daltonics, Billerica, MA, USA) in linear positive mode with a standard 337-nm nitrogen laser operated at 100 Hz. The MALDI IMS acquisition was calibrated initially by using a mixture of standard calibrating proteins: insulin (MW 5,777.6 Da), cytochrome c (MW 12,360.1 Da), and apomyoglobin (MW 16,951.6 Da). Around 200 points in each tissue slice were scanned by 500 consecutive laser shots per point. The flexAnalysis (Bruker Daltonics, Billerica, MA, USA) recorded an average mass spectrum, which mostly presented from 3,000 to 20,000 m/z, with its coordinates on the slice. The flexImaging software (Bruker Daltonics, Billerica, MA, USA) was used for image reconstruction from acquired data.

We used the ClinProTools (CPT) to perform baseline subtraction, normalization, peak calculation, spectral alignment and statistical analyses of data processed by the flexAnalysis. Statistical significance of the peak was analyzed with a Student’s t-test or an ANOVA test on significance levels of 0.05. The CPT reported the Peak statistics, which included the mass, the mass standard deviation (Std), the averaged intensity (Ave), the P value of the t-test/ANOVA (PTTA)^46^ of the individual peak derived from tumor specimen (T) or matched normal tissue (N).

### Immunohistochemistry (IHC) staining analysis of HNSCC paraffin cohort

The HNSCC paraffin sample cohort was obtained from Taipei Medical University Hospital (TMUH), which contains total 86 patients including 79 men and 7 women with 31 NT-paired samples. Their mean age was 53 years, range 30–88 years. 45 patients were diagnosed in early stages (stage I or II) and 41 in late stages (stage III or IV) of HNSCC. Formalin-fixed paraffin embedded tissues (FFPE) were rearranged into a set of serial tissue microarray (TMA) blocks while each sample contained one normal part and three tumor parts derived from different tumor regions to represent its heterogeneity. All participants underwent a series of clinical evaluations then to be treatment according to NCCN guidelines^5^. The clinicopathological data was carefully recorded. The clinical staging was registered based on the American Joint Committee on Cancer (AJCC) TNM Classification of Head and Neck Cancers.

The blocks of FFPE tissue array were sectioned at 3 µm slice thickness. Slides were heated for 1 hr at 60 °C to deparaffinize in xylene, rehydrated in graded alcohol, and rinsed in distilled water. Antigen retrieval and immunohistochemistry staining were performed by using a Benchmark XT automated immunohistochemistry system (Ventana Medical Systems). Tissue array slides were stained with primary TMSB4X antibodies (Proteintech) at 1:400 dilution. Primary antibodies were detected by using an indirect biotin streptavidin system (iVIEW DAB Detection Kit, Ventana Medical Systems, Inc.) and tissue was counterstained in hematoxylin and eosin. Images of stained slides were retrieved by digital scanner (ScanScope Systems Aperio, CA, USA) and scored manually by pathologist, Dr. M Hsiao.

The IHC staining intensity was ranked into bins (0, undetectable; 1, faint; 2, moderate; 3, intense) and represented in Fig. 4A. All cases in an HNSCC cohort (n = 86) were scored by multiplying the intensity (I) and the percentage (P, on a scale of 0% to 100%) which most occupied in each dot region. A final IHC score, i.e. by the so-called quick score (Q)^47^, was presented as Q = P × I; maximum = 300. The three IHC scores of three tumor dots, which were derived from one individual patient, was averaged and assigned as one score. And the score from the matched normal dot, if available, was processed as well.

### Real-time PCR analysis

Total RNA was extracted from tumor and adjacent oral tissues by the acid guanidium-thiocyanate/phenol/chloroform method, and 5 µg of extracted total RNA was subjected to a reverse transcription reaction by following the protocol of reverse transcription kit (Invitrogen). TMSB4X mRNA levels were detected by SYBR realtime PCR (Qiagene). A pair of 5′ primer sequence of TMSB4X is ACAAACCCGATATGGCTGAG while 3′ primer is GAAGGCAATGCTTGTGGA. A pair of 5′ primer sequence of glyceraldehyde-3-phosphate dehydrogenase (GAPDH) is GTCCACTGGCGTCTTCACCACC and 3′ primer is AGGCATTGCTGATGATCTTGAGGC.

### Cell proliferation assay and cell mobility assay

There were 20,000 cells seeded on each well of 6-well plate and the cells were counted every 24 h for 5 days via trypan-blue exclusion method. Cell mobility was evaluated by using a Boyden chamber (Neuro Probe) with 8-µm pore of membrane. Cells were seeded at 15,000 cells per well. For invasion assay, membrane was also coated with 1% thin layer of matrigel (BD). Cells were attracted to move by the serial concentration of FBS in culture medium. Cells were fixed and stained with Giemsa solution (Merck) at 16 hr after seeding.

### Orthotopic xenograft mouse experiments

All animal experiments were performed in accordance with the recommendations in the guidelines for the Care and Use of Laboratory Animals of Academia Sinica. The protocol was approved by the Institutional Animal Care and Use Committee of Academia Sinica (Protocol No: AS-IACUC-15-06-833) with accordance to follow the regulations of replacement and reduction and refinement. The animals were housed in a climate-controlled room (12:12 dark-light cycle, with constant humidity and temperature) and given ad libitum access to food and water. SAS cells were transfected with the luciferase plasmids and stable colonies were selected. The 10^6^ cells were inoculated into left side of buccal mucosa in NSG mouse (SAS-NS group vs. SAS-shTMSB4X group, n = 3 in each group). Tumors on mice were checked weekly by IVIS Spectrum (Xenogen) with intra-peritoneal injection of 150 mg/kg luciferin (Biopath).

### Statistical analysis

Except MALDI IMS spectra, all other spectra were analyzed with FlexAnalysis and ClinProTools (CPT). All spectra were processed by baseline subtraction, peak detection, and peak-area calculation according to default settings. CPT was also used for data visualization, data reduction, and data mining. The classification model of the control and tumor samples was performed by applying multivariate proteomic algorithms, which includes genetic algorithms (GA) and Support Vector Machine (SVM). The prognostic impact of TMSB4X was evaluated by the Kaplan-Meier estimates and compared by the log-rank test. Cox proportional hazards regression model was also performed for calculating the hazard ratio of TMSB4X expression, pathological T and N status. The association between clinicopathological features and TMSB4X IHC expression was analyzed by Pearson’s chi-squared test. For all analyses, A P < 0.05 was considered statistically significant.

## Electronic supplementary material

Supplementary Tables and Figures
