# Supplementary material for: Global Proteomics-based Identification and Validation of Thymosin Beta-4 X-Linked as a Prognostic Marker for Head and Neck Squamous Cell Carcinoma
Source: Sci Rep. 2017 Aug 22;7:9031. doi: 10.1038/s41598-017-09539-w (PMC5567379; doi:10.1038/s41598-017-09539-w)

Supplementary Information

Global Proteomics-based Identification and Validation of Thymosin Beta-4 X-Linked as a Prognostic Marker for Head and Neck Squamous Cell Carcinoma

Li-Hsing Chi[1,2,3], Wei-Min Chang[2], Yu-Chan Chang[2], Yung-Chieh Chan[2], Chia-Chen Tai[2], Kam-Wing Leung[4,5], Chi-Long Chen[6,7], Alexander TH Wu[1], Tsung-Ching Lai[2,*], Yu-Chuan (Jack) Li[1,8,*], and Michael Hsiao[1,2,9,*]

[1]The Ph.D. Program for Translational Medicine, College of Medical Science and Technology, Taipei Medical University and Academia Sinica, Taipei, Taiwan,

[2]Genomics Research Center, Academia Sinica, Taipei, Taiwan

[3]Division of Oral and Maxillofacial Surgery, Department of Dentistry, Taipei Medical University Hospital, Taipei, Taiwan

[4]Department of Density, Yuan's General Hospital, Kaohsiung, Taiwan

[5]Departments of Density, Kaohsiung Veterans General Hospital, Kaohsiung, Taiwan

[6]Department of Pathology, Taipei Medical University Hospital, Taipei Medical University, Taipei, Taiwan

[7]Department of Pathology, College of Medicine, Taipei Medical University, Taipei, Taiwan.

[8]Graduate Institute of Biomedical Informatics, College of Medicine Science and Technology, Taipei Medical University, Taipei, Taiwan

[9]Department of Biochemistry, College of Medicine, Kaohsiung Medical University, Kaohsiung, Taiwan

* Correspondence to Dr. Tsung-Ching Lai, Genomics Research Center, Academia Sinica, Taiwan, E-mail: chuching@gate.sinica.edu.tw; Dr. Yu-Chuan (Jack) Li, Graduate Institute of Biomedical Informatics, College of Medical Science and Technology, Taipei Medical University, Taipei, Taiwan, jaak88@gmail.com; Dr. Michael Hsiao, Genomics Research Center, Academia Sinica, Taiwan, mhsiao@gate.sinica.edu.tw.

Supplementary Table S1. There are 30 proteins identified exclusively in seven HNSCC cell lines. (IPI: international protein index database; * the MASCOT score of the candidate protein)

Accession	Protein ID	Mass	Ca9-22	Cal-27	HSC-3	SAS	SCC-4	TW 1.5	TW 2.6
IPI00024742	Ubiquinol-cytochrome c reductase complex ubiquinone-binding protein QP-C	9769	38	88	67	147	102	102	155
IPI00798268	12 kDa protein	12318	59	200	143	159	104	128	62
IPI00005038	Ribonuclease UK114	14542	184	84	87	85	209	136	102
IPI00795445	17 kDa protein	17203	48	134	64	32	48	142	66
IPI00791107	Protein	18091	59	225	143	159	104	128	62
IPI00791570	Hypothetical protein XRCC5 (Fragment)	18434	62	147	34	68	92	107	61
IPI00291608	Isoform 1 of PEST proteolytic signal-containing nuclear protein	18913	177	238	211	284	205	295	261
IPI00020965	Ubiquitin-conjugating enzyme E2 H	20699	59	48	79	34	35	84	53
IPI00790763	Hypothetical protein HSPD1 (Fragment)	25139	48	356	64	73	48	193	66
IPI00017454	CDNA FLJ19940 fis, clone Y79AA1000833, moderately similar to TUBULIN ALPHA-1 CHAIN	27757	106	146	103	128	44	121	105
IPI00030357	Dihydrogolate reducatase	28940	100	184	96	120	109	115	44
IPI00061108	Isoform 1 of Ribosome recycling factor, mitochondrial precursor	29373	60	88	155	181	141	194	120
IPI00154975	DnaJ homolog subfamily C member 9	30062	155	132	166	402	154	96	95
IPI00001754	Junctional adhesion molecule A precursor	32962	84	55	50	37	53	90	45
IPI00296215	Tumor-associated calcium signal transducer 1 precursor	35582	135	147	94	149	78	102	91
IPI00009659	Uncharacterized protein C20orf77	36991	308	87	42	371	261	139	353
IPI00013468	Mitotic checkpoint protein BUB3	37587	368	235	115	259	165	139	48
IPI00216057	Sorbitol dehydrogenase	38899	108	95	30	30	99	72	92
IPI00026215	Flap endonuclease 1	42908	252	144	139	146	143	209	225
IPI00410693	Isoform 1 of Plasminogen activator inhibitor 1 RNA-binding protein	44995	125	157	121	234	142	166	77
IPI00012535	DnaJ homolog subfamily A member 1	45581	245	235	112	358	198	153	50

IPI00219029	Aspartate aminotransferase, cytoplasmic	46447	294	496	111	220	98	556	277
IPI00217223	Multifunctional protein ADE2	50389	65	132	210	191	281	223	103
IPI00012442	Ras GTPase-activating protein-binding protein 1	52189	112	132	36	128	217	33	86
IPI00552590	chaperonin containing TCP1, subunit 6A isoform b	53711	91	302	223	235	242	269	62
IPI00018873	Isoform 1 of Nicotinamide phosphoribosyltransferase	55772	159	129	40	237	110	135	193
IPI00002214	Importin alpha-2 subunit	58168	327	120	129	217	95	163	121
IPI00784154	60 kDa heat shock protein, mitochondrial precursor	61187	46	1102	277	1841	229	392	267
IPI00179953	NASP Isoform 1 of Nuclear autoantigenic sperm protein	85471	495	222	239	271	109	128	116
IPI00017451	SF3A1 Splicing factor 3 subunit 1	88888	186	66	156	198	108	111	104

Supplementary Table S2. In the correlation analysis of clinicopathological features, the patients with high IHC expression of TMSB4X in nuclei were not significantly associated with those features.

Features	Case no	TMSB4X_nuc				*P* value
		Low(n=38)	(%)	High(n=48)	(%)	
Age at diagnosis						
<65 y	70	30	42.9%	40	57.1%	0.403
>=65 y	16	8	50.0%	8	50.0%	
Gender						
Male	79	35	44.3%	44	55.7%	0.630
Female	7	3	42.9%	4	57.1%	
T Status						
T1+T2	60	23	38.3%	37	61.7%	0.077
T3+T4	26	15	57.7%	11	42.3%	
N Status						
N0	59	27	45.8%	32	54.2%	0.422
N1-N3	27	11	40.7%	16	59.3%	
M Status						
M0	84	37	44.0%	47	56.0%	0.691
M1	2	1	50.0%	1	50.0%	
Clinical Stage						
Stage I+II	45	19	42.2%	26	57.8%	0.434
Stage III+IV	41	19	46.3%	22	53.7%	
Recurrence						
No	32	15	46.9%	17	53.1%	0.435
Yes	54	23	42.6%	31	57.4%	

Supplementary Figure S1. The Venn diagram: A summary of proteins identified in NHOK, OMF and HNSCC by LC-MS/MS.

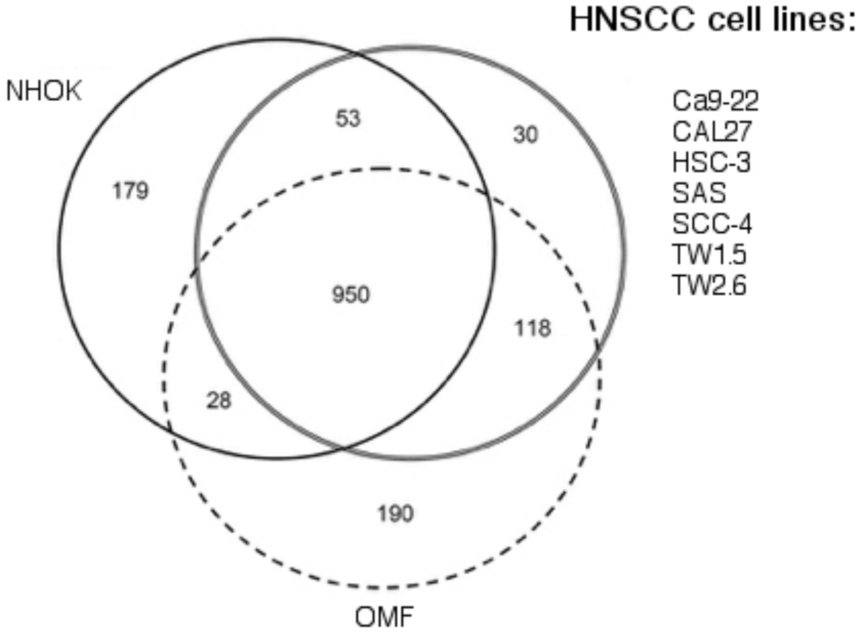

Supplementary Figure S2. The quality control on every tissue array slide of HNSCC cohort. (A) presented the TMSB4X IHC staining as positive staining control from the xenograft tumor of colon cancer cell line, DLD1. (B) showed the negative staining of DLD1 tumor with isotype antibody IgG. (C) and (D) showed the TMSB4X staining of other two colon cancer cell lines, SW620 and Cx-1, respectively.

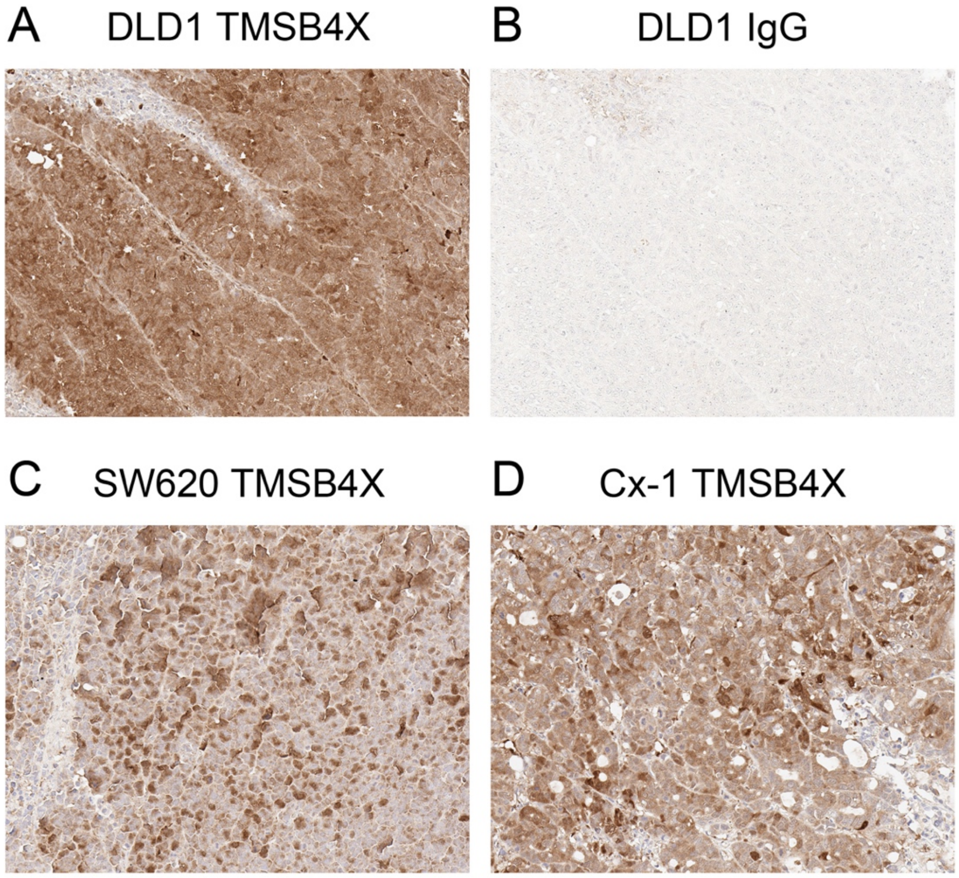

Supplementary Figure S3. The nuclear expression of TMSB4X was not significant correlated with the prognosis in our HNSCC cohort. (A) The representative images with different IHC intensity of TMSB4X in nuclei. (B) And (C) the Kaplan-Meier estimates of OS (P = 0.869) and RFS (P = 0.624) were categorized by nuclear TMSB4X level. Statistics was calculated by log-rank test.

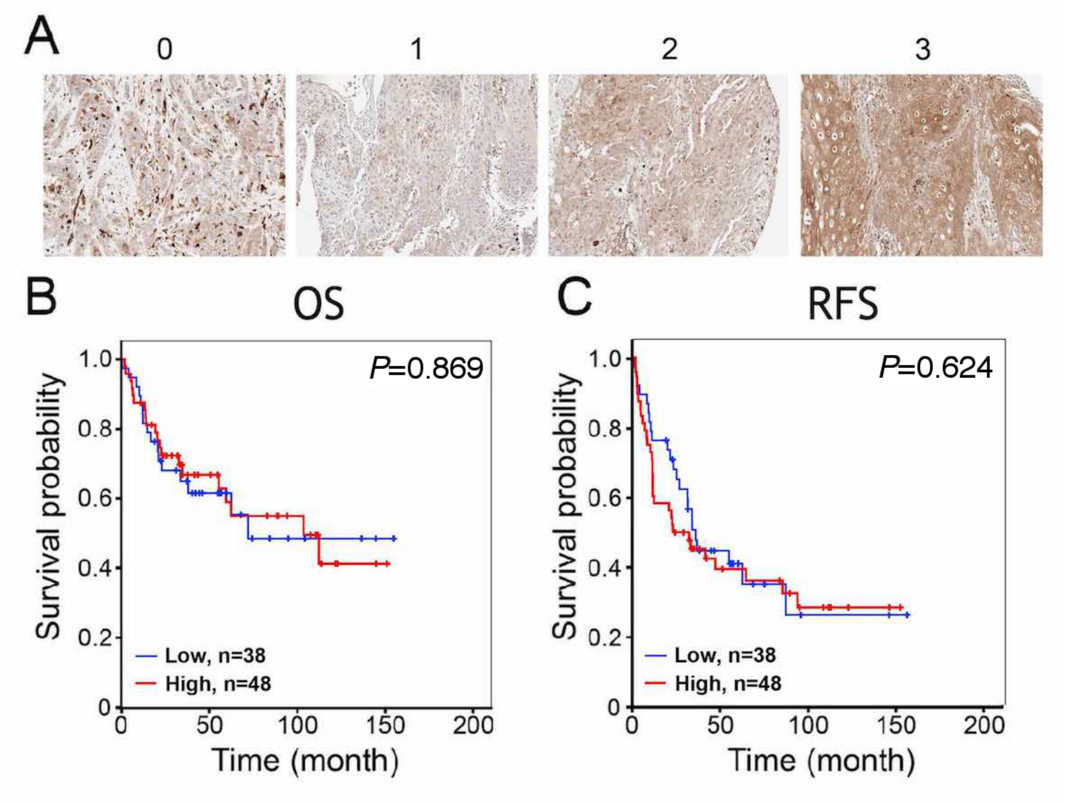

Supplement: Supplementary file 1 — Supplementary Tables and Figures [file 41598_2017_9539_MOESM1_ESM.pdf]
